# Shifted phase of EEG cross-frequency coupling in individuals with Phelan-McDermid syndrome

**DOI:** 10.1186/s13229-020-00411-9

**Published:** 2021-04-28

**Authors:** Michael. G. Mariscal, Elizabeth Berry-Kravis, Joseph D. Buxbaum, Lauren E. Ethridge, Rajna Filip-Dhima, Jennifer H. Foss-Feig, Alexander Kolevzon, Meera. E. Modi, Matthew W. Mosconi, Charles A. Nelson, Craig M. Powell, Paige M. Siper, Latha Soorya, Andrew Thaliath, Audrey Thurm, Bo Zhang, Mustafa Sahin, April R. Levin, the Developmental Synaptopathies Consortium

**Affiliations:** 1 Department of Neurology, Boston Children’s Hospital, Boston, MA USA; 2 Department of Pediatrics, Rush University Medical Center, Chicago, IL USA; 3 Department of Neurological Sciences, Rush University Medical Center, Chicago, IL USA; 4 Department of Biochemistry, Rush University Medical Center, Chicago, IL USA; 5 Seaver Autism Center, Icahn School of Medicine at Mount Sinai Hospital, New York, NY USA; 6 Department of Psychiatry, Icahn School of Medicine at Mount Sinai, New York, NY USA; 7 Department of Genetics and Genomic Sciences, Mount Sinai School of Medicine, New York, NY USA; 8 Department of Neuroscience, Mount Sinai School of Medicine, New York, NY USA; 9 Department of Pediatrics, University of Oklahoma Health Science Center, Oklahoma City, OK USA; 10 Clinical Child Psychology Program, Schiefelbusch Institute for Life Span Studies, University of Kansas, Lawrence, KS USA; 11 Department of Pediatrics, Boston Children’s Hospital, Boston, MA USA; 12 Department of Neurobiology, UAB School of Medicine, Birmingham, AL USA; 13 Department of Psychiatry, Rush University Medical Center, Chicago, IL USA; 14 Intramural Research Program, National Institute of Mental Health, Bethesda, USA

**Keywords:** Phelan-McDermid syndrome, EEG, Power, Cross-frequency coupling, Phase bias

## Abstract

**Background:**

Phelan-McDermid Syndrome (PMS) is a rare condition caused by deletion or mutation of the *SHANK3* gene. Individuals with PMS frequently present with intellectual disability, autism spectrum disorder, and other neurodevelopmental challenges. Electroencephalography (EEG) can provide a window into network-level function in PMS.

**Methods:**

Here, we analyze EEG data collected across multiple sites in individuals with PMS (n = 26) and typically developing individuals (n = 15). We quantify oscillatory power, alpha-gamma phase-amplitude coupling strength, and phase bias, a measure of the phase of cross frequency coupling thought to reflect the balance of feedforward (bottom-up) and feedback (top-down) activity.

**Results:**

We find individuals with PMS display increased alpha-gamma phase bias (*U* = 3.841, *p* < 0.0005), predominantly over posterior electrodes. Most individuals with PMS demonstrate positive overall phase bias while most typically developing individuals demonstrate negative overall phase bias. Among individuals with PMS, strength of alpha-gamma phase-amplitude coupling was associated with Sameness, Ritualistic, and Compulsive behaviors as measured by the Repetitive Behavior Scales-Revised (Beta = 0.545, *p* = 0.011).

**Conclusions:**

Increased phase bias suggests potential circuit-level mechanisms underlying phenotype in PMS, offering opportunities for back-translation of findings into animal models and targeting in clinical trials.

**Supplementary Information:**

The online version contains supplementary material available at 10.1186/s13229-020-00411-9.

## Introduction

Phelan-McDermid Syndrome (PMS) is a well-characterized genetic condition that results from haploinsufficiency of *SHANK3* in the 22q13.3 region. The phenotype in PMS is frequently characterized by intellectual disability [1, 2], autism spectrum disorder (ASD; 50–84%) [3, 4], and epilepsy [5]. *SHANK3* codes for a master scaffolding protein in the postsynaptic density of glutamatergic synapses [6], and its isoforms perform a variety of synaptic functions relevant to neuronal excitability and plasticity [7–15].

A key step in understanding the translational pathway from cells to circuits, networks and ultimately phenotype, involves measurements that reflect large scale network dynamics, including assessments of intrinsic neural oscillations. Electroencephalography (EEG) offers particular opportunity in this regard, because it can measure network dynamics in both humans and animal models, allowing for both forward and back-translation of findings. Clinical EEG (evaluated by visual review) frequently demonstrates abnormalities in PMS, including generalized slowing of activity, slowing or absence of the occipital dominant rhythm, and epileptiform activity [5, 16]. Epileptiform activity on EEG is also frequently seen in ASD more broadly [17]. *Shank3B* null mutant mouse models have demonstrated altered oscillatory power, depending on the location and frequency band studied [9, 14, 18]. Numerous studies in humans with ASD have demonstrated various abnormalities in resting EEG spectral power [19]; however, quantitative studies of EEG activity in humans with PMS have not been previously published.

Recently, there has been increasing interest in the coupling of EEG activity across frequencies, using measures such as Phase-Amplitude Coupling (PAC), given the possibility that such cross-frequency coupling has distinct mechanistic underpinnings. Such coupling is crucial for many of the cognitive functions that are altered in neurodevelopmental disorders, such as long-range communication [20], integration of local and global cortical processing [21], and segmenting and prioritizing sensory input [20, 22]. Altered PAC strength in the alpha-gamma frequency pair has been reported in individuals with ASD at baseline (i.e., rest) and associates with symptom severity [23, 24]. PAC is also altered during tasks in some neurodevelopmental disorders, including during face processing in ASD [25] and cognitive discrimination in a mouse model of Fragile X syndrome [26]. Likewise, PAC during the period preceding an auditory stimulus has been found to positively correlate with non-verbal intelligence quotient in Fragile X syndrome [27]. Cross-frequency coupling thus has theoretical relevance to ASD, intellectual disability, and associated neurogenetic disorders in which such processes are likely altered [28–30].

Recent work suggests not just the strength (the extent to which PAC occurs), but the phase (e.g., where in relation to the alpha waveform gamma amplitude is maximal), can signal important network characteristics. The phase at which fast oscillations are strongest can vary by cortical layer [31] and with alterations in interneuron function [32]. Surface EEG measurements demonstrate the alpha phase resulting in maximum gamma power can vary by age [33] and depth of anesthesia [34]. Differences in PAC phase have been found with encoding success [35] and context [36], and PAC phase bias has been suggested to reflect the ratio of feedforward (bottom-up) to feedback (top-down) cortical activity [33], suggesting the phase of PAC can be functionally relevant particularly among conditions commonly associated with autism spectrum disorder. The timing of gamma within the alpha cycle consequently has the potential to capture alterations in brain connectivity and function that result from specific synaptic perturbations and underlie clinical disorders.

EEG measures of PAC strength and PAC phase thus offer opportunities to enhance understanding of circuit-level dysfunctions in PMS. Here, we first examined whether individuals with PMS, as compared to typically developing (TD) individuals display differences in alpha-gamma PAC strength and phase. Second, we investigated whether these EEG metrics associate with measures of phenotype among individuals with PMS. We hypothesized (1) individuals with PMS would demonstrate increased PAC strength and phase bias, compared to typically developing controls and (2) PAC metrics would correlate with sensory processing difficulties and ASD symptom severity.

## Methods

### Participants

Participants were recruited through a prospective, observational cohort study at four institutions across the United States as a part of the Developmental Synaptopathies Consortium (Clinical Trial NCT02461420): Icahn School of Medicine at Mount Sinai, University of Texas Southwestern, Rush University Medical Center, and Boston Children’s Hospital. Stanford University and the National Institute of Mental Health also participated in the overarching study, but because they only collected phenotyping data and did not collect EEG, participants recruited at those institutions are not included here. In total, 31 individuals with PMS and 17 TD individuals had EEG completed. Participants with PMS were included if they had pathogenic deletions or mutations of the *SHANK3* gene; clinical reports were reviewed to confirm this information. Typically-developing individuals were matched at the group level with PMS participants on chronological age and sex. TD individuals were excluded if they had a diagnosis of any intellectual disability, ASD, or other learning, developmental, psychiatric, or neurological disorders as determined by parent report. All participants were 4 to 19 years of age (inclusive). Informed written consent was obtained from legal guardians and assent was obtained from participants when appropriate. Table 1 shows demographics for participants with adequate EEG data for inclusion (see below).

**Table 1:** Demographic information for participants with useable EEGs

	PMS (n = 26)	TD (n = 15)
Sex	10 (M) 16 (F)	9 (M) 6 (F)
Collection site*		
Icahn School of Medicine at Mount Sinai	11 (42.3%)	10 (33%)
Rush University Medical Center	8 (30.8%)	5 (67%)
Boston Children’s Hospital	7 (26.9%)	0 (0%)
Net type		
Hydrocel 128	18 (69%)	10 (33%)
Biosemi 32	8 (31%)	5 (67%)
Age (years)	9.5 ± 4.25	10.0 ± 2.39
ASD diagnosis		
ASD	11 (42%)	0 (0%)
Non-ASD	14 (54%)	15 (100%)
Unknown	1 (4%)	0 (0%)
ADOS		
Completed	22 (85%)	0 (0%)
Comparison score	6.05 (2.54)	–
Vineland		
Completed	25 (96%)	0 (0%)
Adaptive behavior composite standard score	52.8 ± 13.4	–
Communication composite standard score	58.0 ± 14.6	–
SSP		
Completed	22 (85%)	3 (20%)
Total score	143.9 ± 16.5	178.7 ± 17.1
RBS-R		
Completed	23 (88%)	0 (0%)
Total score	16.26 ± 15.7	–
MSEL		
Completed	16 (61.5%)	0 (0%)
NVIQ	19.98 ± 10.9	–
SB5		
Completed	9 (34.6%)	0 (0%)
NVIQ	46.56 ± 7.5	–
DAS		
Completed	2 (7.7%)	0 (0%)
NVIQ	73.50 ± .71	–
NVIQ		
Completed	25 (96%)	0 (0%)
Non-verbal intelligence quotient	31.4 ± 17.9	–
Seizure history		
Yes	4 (15.4%)	–
No	21 (80.8%)	–
Unknown	1 (3.8%)	15 (100%)
*SHANK3*	–	
Mutation	7 (26.9%)	–
Deletion	19 (73.1%)	–
Deletion size (mega base pairs)	3.95 ± 3.0	–

Categorical variables (i.e. yes, no) are presented as the number in each category, followed by the percentage in each category. Continuous variables are presented as the mean value ± their standard deviation. *No EEGs from University of Texas Southwestern retained enough data after artifact rejection to be analyzed

### Phenotypic data

To examine how our EEG measures related to developmental abilities and ASD phenotypes among individuals with PMS, the following assessments were conducted: the Vineland Adaptive Behavior Scales (Vineland II): Survey Interview Form [37], the Autism Diagnostic Observation Schedule, 2nd edition (ADOS-2) [38], the Autism Diagnostic Interview-Revised [39], the Autism Diagnostic Criteria Checklist from the Diagnostic and Statistical Manual of Mental Disorders, 5th edition [40], the Short Sensory Profile (SSP) [41], and the Repetitive Behavior Scale-Revised (RBS-R) [42]. A psychologist determined ASD diagnosis either on the basis of the study’s assessments or clinical experience when the participant was seen clinically on a regular basis. Additionally, to assess non-verbal cognitive ability, participants were either given the Mullen Scales of Early Learning (MSEL) [43] the Stanford Binet-5 (SB-5) [44], or the Differential Ability Scales, 2nd edition (DAS-II) [45]. Data for a non-verbal intelligence quotient (NVIQ) was compiled depending on the test given: for participants given the SB-5, NVIQ was taken; for participants given the MSEL, the mean of the visual reception developmental quotient (visual reception age equivalent score/age in months) and fine motor developmental quotient (fine motor age equivalent score/age in months) was taken; for participants given the DAS-II, the non-verbal reasoning standard score was taken. Finally, for participants who had experienced seizures, a seizure history was collected.

### EEG acquisition/processing

Continuous EEG was collected for up to 10 min. Participants viewed a silent movie of their choice during EEG recording as is common practice in individuals with neurodevelopmental disorders [47]. EEG was recorded using either a 128-channel Hydrocel Geodesic Sensor Net or a 32 channel ActiveTwo Biosemi net. Data were sampled at either 512 Hz or 1000 Hz (all files were later resampled to 250 Hz). Impedances were kept below the recommendations for the specific EEG system being used prior to recording. For a subset of individuals with PMS, continuous EEG was again collected approximately 12 weeks after the initial recording. These subsequent recordings were used in place of initial recordings if the initial recording did not meet data quality thresholds (n = 1); all other analyses were performed using the initial recording.

Files were processed using the Batch EEG Automated Processing Platform (BEAPP) [48]. Within BEAPP, the Harvard Automated Preprocessing Pipeline for EEG (HAPPE), which was developed specifically to optimize preprocessing of developmental EEG data with potentially high levels of artifact and short recordings, was used to automate preprocessing and artifact minimization [49]. Data were first filtered using a 1 Hz high-pass filter and a 100 Hz low-pass filter. Data were then downsampled to 250 Hz for optimal performance of the HAPPE pipeline. With the exception of Cz, which was used as a reference electrode in some sites’ systems, only electrodes in the international 10–20 system were included in this analysis (18 total) to allow standardization of analyses across net types. Epochs of signal with any channel’s amplitude > 40 μV (the HAPPE default threshold, reflecting the reduced signal amplitude that results from wavelet-thresholding and independent components analysis in HAPPE) were removed. EEG recordings were removed from further analysis if they exceeded thresholds for HAPPE data quality as per [50] in one or more of the following output parameters: percent good channels, mean retained artifact probability, median retained artifact probability, percent of independent components rejected, and percent variance retained after artifact removal. Data were subsequently re-referenced using an average reference, and then segmented into 2 s windows for power and PAC analysis. For each participant, 150 segments (300 s of data) were randomly selected; files with fewer than 150 segments of data at this stage were not analyzed. Primary power and PAC metrics were then obtained using code added to the BEAPP software.

### Power analyses

Power was computed across frequencies using a three taper multitaper window [51]. Power was then computed for a number of frequency bands: Delta [1–4 Hz), Theta [4–8 Hz), Alpha [8–12 Hz), Beta [12–30 Hz), and Gamma [30–55 Hz). Total power was computed as all frequencies between [1–55 Hz]. The power at each frequency band and the overall 1–55 Hz range was computed by adding the power spectral density over the frequency range of interest.

To capture each frequency band’s relative contribution to total power, the relative power at each frequency band was computed as the power at each frequency band divided by the total power. Power values were then averaged across electrodes. Visual inspection of the power spectra, averaged across the occipital channels analyzed in this study (O1 and O2), was used to identify the peak alpha frequency of each participant.

### PAC analysis

#### Modulation index

To capture the presence of coupling, PAC was first quantified using the Modulation Index (MI) [52]. Because the data are not time locked to any specific task, we focus on PAC in the alpha-gamma range, where prior studies have shown abnormalities in other neurodevelopmental disorders using resting or non-time-locked data [23, 24]. For each frequency pair, the raw signal in each segment was exported from MATLAB into Python and filtered into a range of alpha (8–12 Hz in 2 Hz steps) and gamma (here, 28–56 Hz to allow for division into 4 Hz steps) frequencies using code adapted from Dupré la Tour et al. [53]. Alpha frequencies were filtered using a constant bandwidth of 2 Hz, while gamma frequencies were filtered using an upper sideband variable bandwidth, so as to avoid including phase frequencies in the amplitude frequencies. In detail, for each gamma frequency, the lower passband cutoff was 2 Hz below the gamma frequency, and the upper passband cutoff was set as the alpha phase frequency plus the gamma amplitude frequency [24]. For example, for the combination of 40 Hz gamma and 8 Hz alpha, the lower limit of the gamma amplitude filter was 38 Hz (40 − 2), while the upper limit of the filter was 48 Hz (40 + 8). Filtering at this step consisted of a zero-phase cosine-based filter to extract the real component, and then a sine-based filter to extract the imaginary component, resulting in a complex-valued output signal [53]. The alpha phase time series, or gamma amplitude time series, were obtained from this complex signal. The phases of the alpha signal were then binned into 18 20° intervals (− 180° to 180°), and the mean of the amplitude of the gamma signal occurring within each phase bin was calculated. Mean gamma amplitude values in each phase bin were then normalized by dividing each bin value by the sum of all bin values. Data were then imported into MATLAB, where the amplitude of the gamma signal at each phase bin of the alpha signal was then averaged together across segments. The MI_raw_ was then computed as the Kullback–Leibler divergence of the gamma amplitude distribution from a uniform distribution [52]. We then employed a time-shift procedure to control for factors that may generate spurious phase-amplitude coupling. In detail, for each participant, 200 surrogate MI values (MI_surr_) were generated by repeating the procedure after offsetting gamma amplitude from the alpha phase distribution by a randomized time shift between 0.1 and 1.9 s. A normalized MI (z-MI) was then computed as the z-score of the MI_raw_ compared to the distribution of MI_surr_ values [54]. The z-MI at each alpha and gamma frequency combination was then averaged to obtain a single overall alpha-gamma PAC value for each participant, at each electrode.

#### Phase bias

Modulation Index captures the extent of coupling. We additionally set out to quantify whether gamma amplitude increased closer to the rising or falling phase of the alpha waveform, and to what degree. To do so, we employed a metric termed phase bias, drawing on measures of phase preference [32] and prior findings that this tends to show a bimodal distribution (i.e., with fast activity occurring maximally at either the negative or positive phases (corresponding to rising and falling phases, respectively) of the slower waveform [35]. Specifically, we quantified the phase bias of the gamma amplitude to the positive phases of the alpha waveform; i.e., the relative change of gamma amplitude (gamma_amp_) during the positive phases (0°–180°) of the alpha waveform. Thus, phase bias is calculated as (Σgamma_amp_ in positive phases of the alpha waveform)/Σ(gamma_amp_ in all phases of the alpha waveform) − 0.5. Importantly, here, a cosine-based filter was used to extract alpha phase; as a result, 0° corresponds to the peak of alpha, + 90° corresponds to the falling zero crossing of alpha, 180°/− 180° corresponds to the trough of alpha, and − 90° corresponds to the rising zero crossing of alpha. Therefore, a phase bias > 0 indicates gamma amplitude increases at the falling phase of the alpha waveform, and a phase bias < 0 indicates gamma amplitude increases at the rising phase of the alpha waveform. Additionally, a larger distance from 0 (where gamma amplitude does not increase preferentially at either positive or negative phases of alpha) indicates stronger phase bias. The phase bias at each alpha frequency and gamma high frequency combination was then averaged to obtain a single overall alpha-gamma phase bias value.

### Statistical analysis

#### Group comparisons

We first set out to test whether power or phase-amplitude coupling metrics differed between groups. Because most metrics were not normally distributed, all group comparisons were performed using a non-parametric test (independent samples Mann–Whitney U) unless otherwise specified. Relative power in each frequency band was compared between groups, and an independent samples *t* test was used to compare peak alpha frequency between groups. To test whether overall PAC metrics differed in individuals with PMS as compared to typically developing individuals, group comparisons were first performed on z-MI and phase bias data averaged across all 10–20 electrodes. Subsequently, because PAC has been shown to differ between anterior and posterior scalp areas [33], these group comparisons were repeated after averaging PAC metrics across all anterior 10–20 electrodes (Fp1, Fp2, F3, F4, F7, F8, Fz) and then posterior 10–20 electrodes (P3, P4, P7, P8, Pz, O1, O2). Finally, these comparisons of overall, anterior, and posterior z-MI and phase bias were repeated between individuals with PMS diagnosed with ASD (N = 11), and individuals with PMS diagnosed without ASD (N = 14). Data were analyzed in SPSS (IBM Corp, 2016).

#### Clinical associations

All associations were performed using linear regression analysis. Because PAC has been shown to change with age [33], we tested whether age was associated with PAC metrics among all participants. Additionally, to test whether the relationship between ln(z-MI) and age was different in individuals with PMS as compared to TD individuals, a regression was performed, with ln(z-MI) as the dependent variable, and age, group, and age by group included as independent variables. The association between alpha power and PAC metrics (averaged across all electrodes) was additionally examined. To test how PAC associated with behavioral phenotype in individuals with PMS, linear regression analysis was performed between PAC metrics (z-MI and phase bias) and the following measures: Vineland Adaptive Behavior Composite, Vineland Socialization Composite, ADOS comparison score, SSP, RBS-R, and NVIQ. Additional linear regressions were performed between z-MI and the 6 behavior sub-scales of the RBS-R (Restricted Interest, Sameness, Ritualistic, Compulsive, Self-Injurious, and Stereotypic). Because z-MI did not demonstrate a normal distribution, linear regressions were performed on the natural log transformation of z-MI; one negative z-MI value was not included in this analysis. Age was included as a control variable in all regressions.

#### Clinical comparisons

In individuals with PMS, we tested whether PAC measures differed by a number of categorical clinical variables, including: sex, presence of an ASD diagnosis, presence of a seizure history (at least one seizure event experienced), and whether the participant has a *SHANK3* mutation or deletion. All comparisons were computed using a Mann–Whitney U test. For all associations between EEG and clinical measures, a Benjamini–Hochberg correction was applied to power, ln(z-MI) and phase bias clinical correlations separately (FDR = 0.1).

### Participants with insufficient EEG data

In total, there were 33 individuals with PMS and 17 TD individuals in the study. After removal of participants who did not complete EEG (n = 2 with PMS), had insufficient data quality (n = 4 with PMS, n = 1 TD), or had insufficient data length (n = 1 with PMS and n = 1 with TD), 26 individuals with PMS and 15 TD individuals remained for further analysis. Compared to PMS participants included in this dataset, the 7 PMS participants excluded for unusable EEG data were more likely to be male (6/7). Otherwise, they were not significantly different in age (mean = 9.94, SD = 4.58, *p* = 0.7587), they demonstrated similar prevalence of ASD diagnosis (4/7) and seizure history (2/7) and there were no significant differences in included vs. excluded participants with PMS on the Vineland Adaptive Behavior Composite, the Vineland Communication Composite, ADOS severity score, NVIQ, SSP, or RBS-R (*p* > 0.05). All tests were performed using an independent samples Mann–Whitney U test.

## Results

### Power and phase-amplitude coupling in PMS

**Fig. 1:**
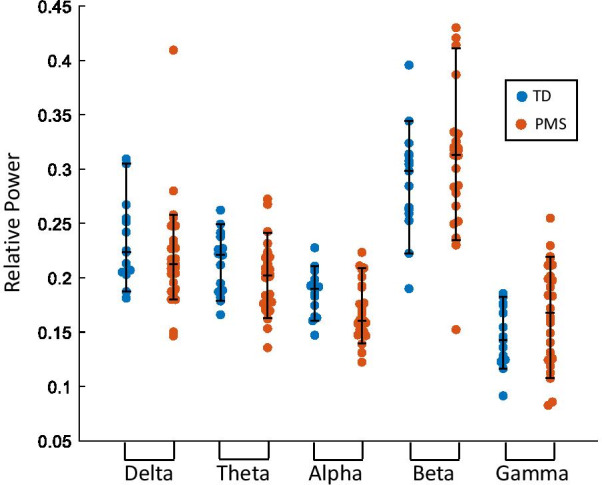
Relative power values over all electrodes in the 10–20 system at each frequency band of TD individuals compared with individuals with PMS. Median, 10th and 90th percentiles are plotted

In all frequency bands tested, no differences in relative power were observed between individuals with PMS and TD individuals after correcting for multiple comparisons (FDR = 0.1, 5 power comparisons) (Fig. 1). Reduced alpha power in PMS relative to TD approached, but did not reach, significance (*U* = − 4.457, *p* = 0.035). Gamma power enhancements in PMS also did not reach significance (*U* = 2.380, *p* = 0.123).

Mean peak alpha frequency was found to be 9.38 Hz (SD = 0.921 Hz) in individuals with PMS, and 9.43 Hz (SD = 0.610) in TD individuals; peak alpha frequency was not found to differ between groups (*t* = 0.170, *p* = 0.866). Two individuals with PMS and two TD individuals did not display a peak in alpha activity, and two individuals with PMS demonstrated a peak alpha frequency outside of the 8–12 Hz range (7.32 and 7.81 Hz). These participants all displayed PAC values in the typical range.

**Fig. 2:**
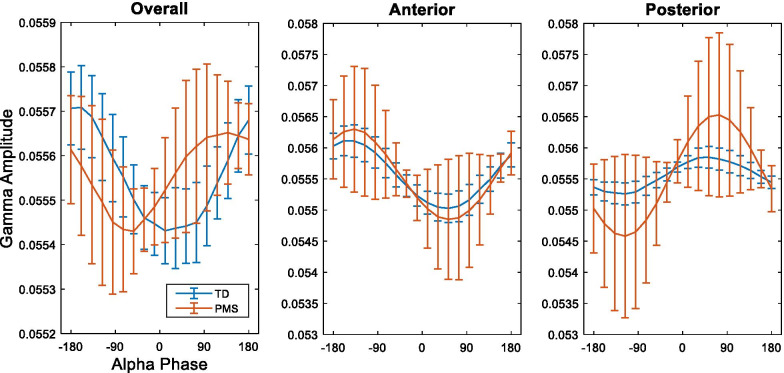
Gamma (28–56 Hz) amplitude plotted as a function of alpha (8–12 Hz) phase, in all channels (left), anterior channels (middle), and posterior channels (right). For each group, amplitude mean and standard deviation values are plotted

**Fig. 3:**
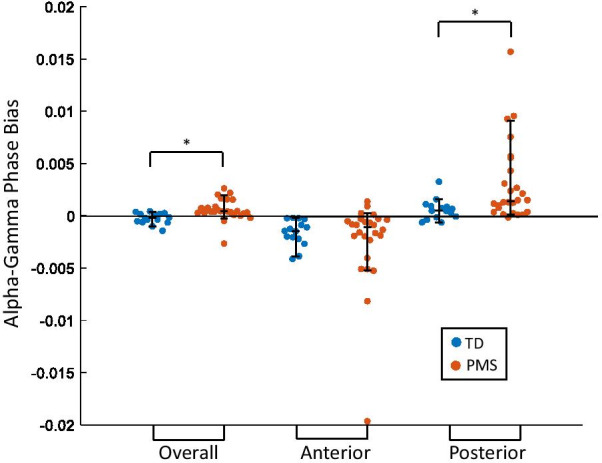
Alpha (8–12 Hz)–Gamma (28–56 Hz) phase bias values of TD individuals compared to individuals with PMS. Comparisons were done using phase bias values averaged across all channels (Overall), all anterior channels (Anterior), and all posterior channels (Posterior). Median, 10th and 90th percentiles are plotted. *Indicates significance at Benjamini–Hochberg corrected *p* value of .0167

When averaging across all channels, individuals with PMS largely demonstrated maximal gamma amplitude at the falling phase of the alpha cycle, whereas TD participants largely demonstrated maximal gamma amplitude at the rising phase of the alpha cycle (Fig. 2). Consequently, individuals with PMS demonstrated a positive overall phase bias (median = 4.091 * 10^–4^, SD = 1.02 * 10^–3^), and TD individuals demonstrated a negative overall phase bias (median = − 2.079 * 10^–4^, SD = 5.29 * 10^–4^) (Fig. 3). After correcting for multiple comparisons (FDR = 0.1, 6 PAC comparisons), overall phase bias was significantly greater in individuals with PMS than TD individuals (*U* = 3.519, *p* = 0.0005).

**Fig. 4:**
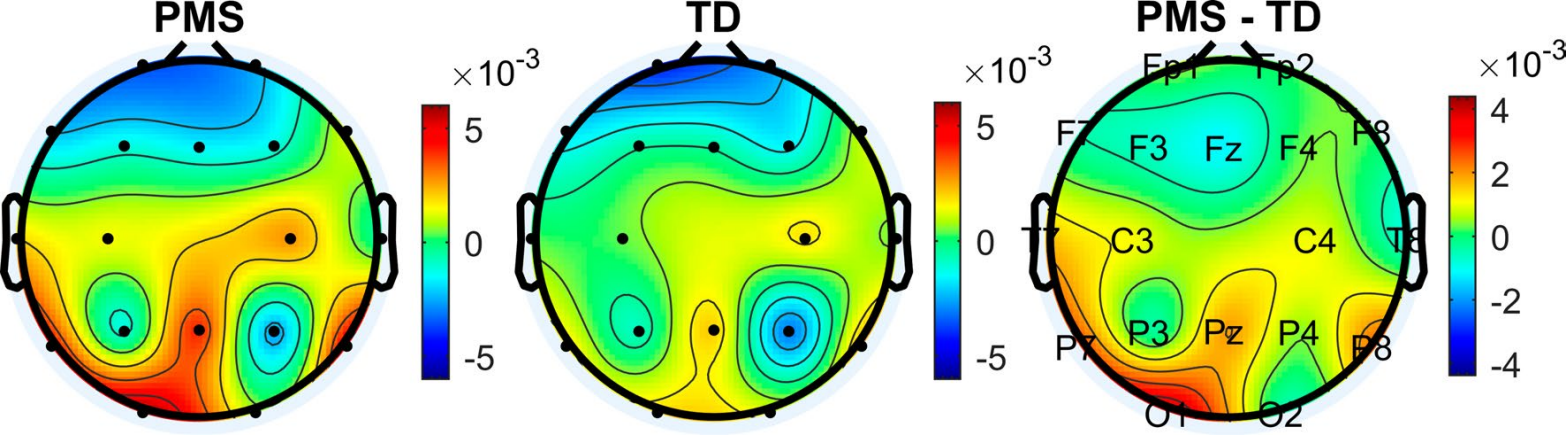
Topographies of Alpha (8–12 Hz)–Gamma (28–56 Hz) phase bias values. The mean phase bias values for each group are shown, as well as the difference between the group means

**Fig. 5:**
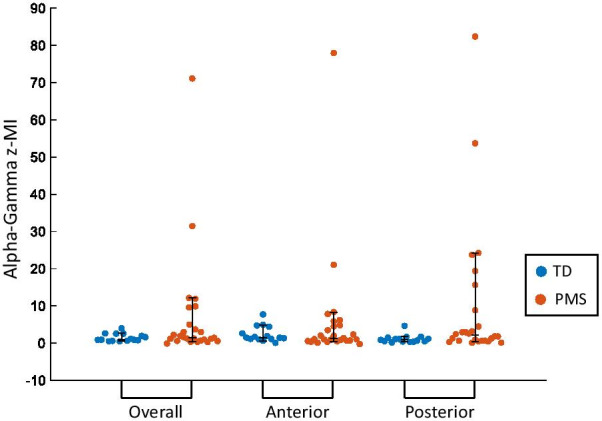
z-MI values of Alpha (8–12 Hz)–Gamma (28–56 Hz) coupling in TD individuals compared to individuals with PMS. Comparisons were done using z-MI values averaged across all channels (Overall), all anterior channels (Anterior), and all posterior channels (Posterior). Median, 10th and 90th percentiles are plotted

In both TD individuals and individuals with PMS, phase bias largely increased along the anterior–posterior axis of the scalp, with the exception of electrodes P3 and P4, which demonstrated a negative phase bias in both groups (Fig. 4). Analysis of phase bias in anterior and posterior regions separately showed posterior electrodes exhibited increased phase bias in individuals with PMS (*U* = 2.734, *p* = 0.006) while anterior electrodes did not exhibit a difference between groups (*U* = 0.189, *p* = 0.862) (Figs. 2, 3). Likewise, posterior z-MI was significantly increased in individuals with PMS (*U* = 2.165, *p* = 0.030), while anterior z-MI did not demonstrate a difference between groups (*U* = − 0.352, *p* = 0.738). On the other hand, no differences were found when comparing overall z-MI between groups (Fig. 5, Additional file 1: Table S1). In all participants, no association was found between alpha power and ln(z-MI) (Beta = 0.156, *p* = 0.337), or phase bias (Beta = − 0.240, *p* = 0.131) when averaging across all electrodes.

### Phase-amplitude coupling and clinical characteristics

**Fig. 6:**
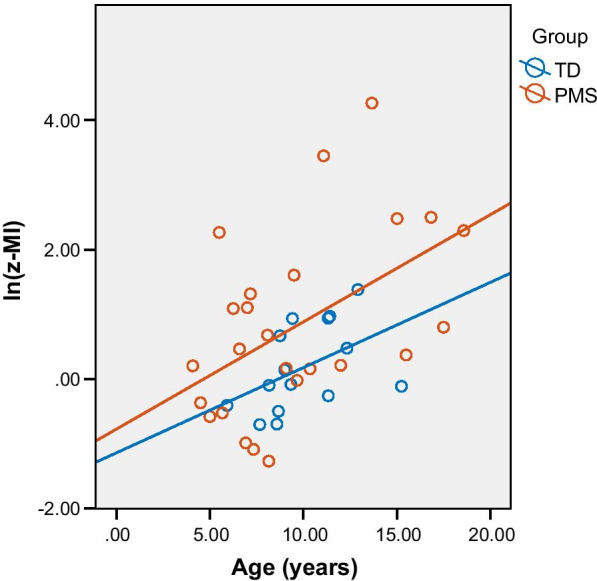
Relationship between age and ln(z-MI). Trend lines are plotted separately for typically developing individuals and individuals with PMS

In all participants, age was significantly associated with ln(z-MI) (Beta = 0.465, *p* = 0.002); age was therefore controlled for in subsequent regressions (Table 2). In a regression comparing ln(z-MI) and age, the interaction between age and group was not significant (Beta = 0.017, *p* = 0.916), indicating the relationship between age and ln(z-MI) does not differ between individuals with PMS and TD individuals (Fig. 6). In individuals with PMS, ln(z-MI) increased with RBS-R total score (Beta = 0.545, *p* = 0.011); specifically, ln(z-MI) was found to increase with the Sameness, Ritualistic, and Compulsive sub-scales of the RBS-R (Table 3). No other associations between PAC measures and phenotypic measures reached significance. Additionally, no significant differences in PAC metrics between categorical phenotypic variables within individuals with PMS (sex, ASD diagnosis, *SHANK3* deletion, seizure history) were observed (Table 4).

**Table 2:** Associations between behavioral phenotype and PAC metrics in Phelan-McDermid syndrome

Measure	Standardized beta coefficient	*P* value
ln(z-MI) versus	–	–
Age	.500	.011*
Vineland adaptive behavior	.146	.555
Vineland socialization	− .040	.870
Deletion size	− .092	.723
SSP total	− .336	.225
ADOS comparison score	− .379	.150
RBS-R total score	.545	.011*
NVIQ	.618	.543
Phase bias versus	–	–
Age	.194	.342
Vineland adaptive behavior	− .104	.630
Vineland socialization	− .202	.343
Deletion size	.232	.261
SSP total	.367	.100
ADOS comparison score	.026	.907
RBS-R total score	− .092	.652
NVIQ	− .980	.338

All tests were performed as linear regressions, with age included as a control variable for all subsequent tests. A Benjamini–Hochberg correction was applied to ln(z-MI) and phase bias comparisons separately. * indicates significance (FDR = .1)

**Table 3:** Associations between RBS-R sub-scales and ln(z-MI) in Phelan-McDermid Syndrome

RBS-R sub-scale	Standardized beta coefficient	*P* value
Restricted interest	.337	.183
Sameness	.546	.012*
Ritualistic	.490	.019*
Compulsive	.448	.038*
Self-injurious	.206	.374
Stereotypic	.362	.153

All tests were performed as linear regressions, with age included as a control variable for all subsequent tests. A Benjamini–Hochberg correction was applied. * indicates significance (FDR =.1)

**Table 4:** Comparing PAC metrics across categorical phenotypes in Phelan-McDermid Syndrome

Measure	PAC median	Mann–Whitney *U*	*P* value
z-MI	–	–	–
Sex	–	–	–
Female	2.99 (1.19, 9.83)	2.16	.031
Male	0.836 (0.512, 1.485)	–	–
ASD diagnosis	–	–	–
Yes	1.45 (0.337, 3.73)	-.712	.501
No	1.60 (0.907, 6.71)	–	–
* SHANK3*	–	–	–
Deletion	1.22 (0.559, 4.98)	− .723	.497
Mutation	1.59 (1.23, 9.63)	–	–
Seizure history	–	–	–
Yes	1.84 (.086, 9.50)	.371	.748
No	1.23 (.626, 4.35)	–	–
Phase bias * 10^4^	–	–	–
Sex	–	–	–
Female	5.00 (2.29, 15.7)	1.00	.336
Male	3.67 (− 0.007, 5.17)	–	–
ASD diagnosis	–	–	–
Yes	4.28 (2.88, 15.5)	.876	.403
No	3.33 (.534, 7.85)	–	–
* SHANK3*	–	–	–
Deletion	4.14 (0.210, 15.7)	.145	.910
Mutation	4.04 (0.751, 7.57)	–	–
Seizure history	–	–	–
Yes	7.99 (− 1.85, 18.5)	.889	.409
No	3.84 (1.42, 6.71)	–	–

Median values (25th and 75th percentile values in parentheses) are presented for each category. All tests performed using a Mann–Whitney *U* test. A Benjamini–Hochberg correction was applied to ln(z-MI) and phase bias comparisons separately. * indicates significance (FDR = .1)

## Discussion

We find individuals with PMS show significantly increased alpha-gamma phase bias relative to TD individuals, with most individuals with PMS demonstrating positive overall phase bias, whereas most typically developing individuals demonstrated negative overall phase bias in our sample. Between-group differences are primarily driven by findings over posterior electrodes, where phase bias and PAC are both more strongly positive in individuals with PMS relative to TD individuals. Previous work has reported greater alpha-gamma PAC in a midline parietal–occipital source in individuals with ASD [24]. Within individuals with PMS, no differences were observed with measures of overall ASD phenotype, or social functioning; however, RBS-R total score was found to increase with increased PAC strength, indicating in individuals with PMS, PAC strength may map on to this aspect of the ASD symptom profile specifically.

The between-group differences in phase bias suggest that circuit function is perturbed in PMS, in a manner measurable by surface EEG. This finding suggests several opportunities for back-translation into animal models to elucidate underlying mechanisms. For example, scalp level EEG does not reflect the unified activity of the cortex, but rather the grand average of many networks often exhibiting conflicting activity. Phase bias is known to vary by cortical layer. Laminar recordings in monkeys and rats demonstrate that spontaneous current sinks in theta and alpha bands in layers 2/3-5a are associated with high gamma amplitudes and high action potential firing (and sources are associated with low gamma amplitudes and low action potential firing) whereas the opposite is true in layer 6 (sinks are associated with low gamma amplitudes and low action potential firing, and sources with high gamma amplitudes and high action potential firing) [31, 55]. Additionally, alpha current generators in layers 2/3 and 6 are in phase with one another, but out of phase with those in layer 4 [55], meaning whether scalp-level EEG gamma activity is phase-locked to the falling phase or the rising phase of alpha could depend on whether alpha activity from layers 2/3 and 6 or layer 4 dominates the signal. Therefore, it is possible the phase bias presented here depends on the relative PAC and alpha activity of each cortical layer. Cortical layer 4 predominantly accepts feedforward (thalamocortical) input, layer 6 predominantly provides feedback (corticothalamic output), and layer 2/3 integrates feedforward, feedback, and lateral activity [56, 57]. Between-group differences in surface level phase bias may therefore suggest altered balance of feedforward versus feedback information transfer in PMS; this could be further examined in animal models.

Here, the phase bias abnormalities in individuals with PMS were localized to electrodes over the posterior cortex. Alpha-gamma PAC has been previously shown to increase in the occipital cortex during visual tasks [58]. Notably, the present study analyzed EEG recordings collected while participants watched a silent movie. Cases of cortical visual impairment have been reported in some individuals with PMS [59]; therefore, the network perturbations captured here may also relate to abnormalities in visual processing in PMS.

Notably, we did not identify any differences in EEG power, in any frequency band, between individuals with PMS and TD individuals. This is in contrast to prior electrophysiological measurements in animal models, where power differences have been demonstrated in specific regions and frequency bands [9, 14, 18]. Still, no consistent findings have emerged in studies of animal models as well as in clinical studies. Here, we used relatively conservative statistical techniques, and a slight relaxing of statistical thresholds would have led to findings of overall low alpha power and high gamma power, consistent with some prior animal studies [9, 14]. Nonetheless, the PAC effects (particularly phase bias) are quite strong and persist despite these conservative techniques. While prior studies of PAC during some tasks demonstrate an inverse relationship between alpha power and PAC [60], we did not identify any such relationship in our sample. This suggests that our PAC findings are not driven by changes in nonsinusoidal alpha activity, and that PAC and alpha power can be independently modulated.

By grossly reflecting neural network activity, EEG is an intermediary on the spectrum from genotype to phenotype. Given the myriad possibilities for analysis that EEG offers, EEG itself thus also reflects a smaller spectrum-within-a-spectrum from genotype to phenotype, depending on the exact analysis chosen. Here we demonstrate that one analytic technique (phase bias) leans toward a reflection of genotype more than phenotype. Notably, we do not have any data to suggest that this phase bias anomaly is specific to PMS; in fact, it is quite possible that similar phase bias anomalies could be present in other genetic disorders that affect similar pathways (e.g., other disorders of the mTOR pathway), and further research is necessary to test this. On the other hand, our findings suggest that zMI likely measures an aspect of neural network function that leans more towards phenotype; therefore, future research should explore whether zMI anomalies are associated with restricted and repetitive behaviors in other neurodevelopmental disorders. Along similar lines, comparison of PAC and z-MI findings between PMS and a phenotypically similar cohort would also be of interest.

However, the genotype–phenotype spectrum is just one of many axes that EEG-based measures may reflect. For example, EEG-based measures may also change across development. We find that z-MI increases with age in our sample (mean age 9.7 years), but phase bias does not. This extends prior findings, in which z-MI was found to increase across the first 3 years after birth in typical development [33]. Developmental effects are particularly important to consider in PMS given the known molecular and electrophysiological functions of *SHANK3*, including effects on plasticity*.* SHANK3 provides scaffolding in the postsynaptic density of glutamatergic synapses [6], and *Shank3* mutant mouse models have therefore demonstrated decreased excitability of glutamatergic [8, 9, 14, 15] and GABAergic neurons [11]. Plasticity is also impaired in *Shank3* mutants [13]. Excitability can be altered by developmental activity and plasticity within circuits, at times leading to seemingly contradictory findings. For example, when inhibition is impaired more than excitation within corticostriatal circuitry during early development, the balance between activity of excitatory and inhibitory neurons can lead to cortical hyper-activity, with resulting changes in plasticity that ultimately cause high (rather than low) excitability of GABAergic neurons in this circuit [12]. In layer 2/3 of primary somatosensory cortex, *Shank3* deficiency causes decreased excitability of GABAergic interneurons but increased excitability of glutamatergic neurons [11]. Trajectories across development, combined with studies examining primary and compensatory mechanisms underlying these trajectories, can provide additional clues about the biological underpinnings of neurodevelopmental disorders including (but not limited to) PMS.

### Limitations

Our ability to detect subtle phenotypic associations was hampered by several limitations. First, as is common in rare disease research, the sample size in the PMS group led to limited statistical power for assessing associations with categorical variables within this group. In particular, only 4 PMS individuals exhibited a history of seizures. Additionally, our typically developing cohort was small (15 EEGs analyzed), limiting our ability to identify differences with the PMS group. Second, the severity of PMS led several behavioral measures to suffer from a ‘floor’ effect, making it difficult to compare the phenotypes of individuals within the PMS group. Clinical assessments were also rarely conducted in in the typically developing cohort; as a result, the present study was not able to test for associations between PAC measures and clinical variables in this group. Also of note, though the individuals with PMS enrolled in this study that were not able to provide adequate EEG data for analysis did not demonstrate clear differences on phenotyping measures, they do represent a subgroup of individuals with PMS this study was not able to capture. Finally, though the scalp-level EEG used here allows us to describe differences in grand-average oscillatory activity, it is unable to differentiate the specific neural mechanisms underlying these differences; back-translation into animal models will likely be necessary to further explore this.

## Conclusion

Altered phase bias in PMS suggests altered network dynamics in this disorder. Mechanisms underlying altered network activity in PMS can be further elucidated using back-translation to determine underpinnings of phase bias abnormalities in animal models.

Future studies may assess the extent to which individuals with other neurodevelopmental and neurogenetic disorders have altered phase bias similar to that seen in PMS, suggesting common underlying mechanisms at the network level, and the extent to which phase bias may change in response to treatment in clinical trials.

## Supplementary Information

**Additional file 1.** **Supplementary Table 1**. Group comparisons for power in each frequency band, and phase-amplitude coupling metrics. Median values (25th and 75th percentile values in parentheses) are presented for each category. All tests performed using a Mann-Whitney U test. A Benjamini-Hochberg correction was applied to power and PAC separately. * indicates significance (FDR = .1)

## Data Availability

The datasets analyzed during the current study are available from the corresponding author on reasonable request.

## Abbreviations

PMS: Phelan-McDermid syndrome
ASD: Autism spectrum disorder
EEG: Electroencephalography
PAC: Phase-amplitude coupling
TD: Typically developing
ADOS: Autism Diagnostic Observation Schedule
SSP: Short sensory profile
RBS-R: Repetitive Behavior Scale-Revised
MSEL: Mullen scales of early learning
SB-5: Stanford Binet-5
DAS-II: Differential ability scales, 2nd edition
NVIQ: Non-verbal intelligence quotient
BEAPP: Batch EEG Automated Processing Platform
HAPPE: Harvard Automated Preprocessing Pipeline for EEG
MI: Modulation index
z-MI: Z-scored Modulation Index
FDR: False discovery rate
