# Supplementary material for: Shifted phase of EEG cross-frequency coupling in individuals with Phelan-McDermid syndrome
Source: Mol Autism. 2021 Apr 28;12:29. doi: 10.1186/s13229-020-00411-9 (PMC8082621; doi:10.1186/s13229-020-00411-9)
Supplement: Supplementary file 1 — Additional file 1. Supplementary Table 1. Group comparisons for power in each frequency band, and phase-amplitude coupling metrics. Median values (25th and 75th percentile values in parentheses) are presented for each category. All tests performed using a Mann-Whitney U test. A Benjamini-Hochberg correction was applied to power and PAC separately. * indicates significance (FDR = .1) [file 13229_2020_411_MOESM1_ESM.docx]

**Supplementary Material**

| Frequency Band | TD | PMS | Mann–Whitney *U* | P value |
| --- | --- | --- | --- | --- |
| Delta | .224 (.205, .255) | .213 (.189, .238) | -.949 | .330 |
| Theta | .221 (.187, .238) | .202 (.175, .219) | -2.297 | .130 |
| Alpha | .190 (.164, .199) | .161 (.150, .192) | -4.457 | .035 |
| Beta | .298 (.259, .314) | .313 (.263, .327) | 1.114 | .291 |
| Gamma | .143 (.125, .175) | .168 (.125, .204) | 2.380 | .123 |
| Overall |  |  |  |  |
| z-MI | .919 (.664, 2.545) | 1.520 (.667, 6.140) | 1.191 | .242 |
| Phase Bias *10^4^ | -2.079 (-6.151, 1.740) | 4.091 (1.760, 10.404) | 3.519 | .0005* |
| Anterior |  |  |  |  |
| z-MI | 1.479 (1.103, 4.414) | 1.220 (.649, 5.086) | -.352 | .738 |
| Phase Bias *10^4^ | -14.587 (-22.143, -3.222) | -11.150 (-27.520, -3.517) | .189 | .862 |
| Posterior |  |  |  |  |
| z-MI | .892 (.424, 1.481) | 2.132 (.664, 10.538 | 2.165 | .030* |
| Phase Bias *10^4^ | 4.846 (-1.471, 9.241) | 13.885 (3.545, 46.073) | 2.734 | .006* |

**Supplementary Table 1.** Group comparisons for power in each frequency band, and phase-amplitude coupling metrics. Median values (25^th^ and 75^th^ percentile values in parentheses) are presented for each category. All tests performed using a Mann-Whitney *U* test. A Benjamini-Hochberg correction was applied to power and PAC separately (FDR = .1).
